# Patient satisfaction and safety in the administration of sedation by nursing staff in the digestive endoscopy service: a cross-sectional study

**DOI:** 10.1186/s12912-024-02644-y

**Published:** 2024-12-28

**Authors:** Miriam Hidalgo-Cabanillas, José Alberto Laredo-Aguilera, Ana Isabel Cobo-Cuenca, Rosa María Molina-Madueño, Esmeralda Santacruz-Salas, Pedro Manuel Rodriguez-Muñoz, Juan Manuel Carmona-Torres

**Affiliations:** 1 Hospital Universitario de Toledo, Toledo, 45004 Spain; 2 Facultad de Fisioterapia y Enfermería, Universidad de Castilla-La Mancha, Toledo, 4507 Spain; 3 Grupo de Investigación Multidisciplinar en Cuidados (IMCU), Universidad de Castilla-La Mancha, Toledo, 45071 Spain; 4 Instituto de Investigación Sanitaria de Castilla-La Mancha (IDISCAM), Toledo, 45004 Spain; 5 Hospital Universitario Rey Juan Carlos, Mostoles, 28933 Spain

**Keywords:** Nurse anesthetists, Endoscopy, Nursing, Patient satisfaction, Sedation, Endoscopy digestive sistem

## Abstract

**Background:**

The satisfaction of patients with sedation by nursing staff is an issue of interest for the quality of health care, influencing the recovery and well-being of patients as well as their confidence in and adherence to treatment. One of the most frequently performed diagnostic and therapeutic tests requiring sedation are digestive endoscopies, so it would be interesting to study satisfaction in these services.

**Aim:**

To determine the level of patient satisfaction and safety with sedation via digestive endoscopies by nurses.

**Methods:**

This was a cross-sectional study in the Digestive Endoscopy Service at the University Hospital of Toledo, Spain. The sample consisted of 660 adult patients from the digestive endoscopy service who were sedated between June–September 2023. The degree of satisfaction with the service was measured by the questionnaire: Survey of patient satisfaction with the digestive endoscopy service. The study was approved by the ethics committee.

**Results:**

Patients who reported satisfaction with the treatment were very satisfied with the sedation provided by the nurses. The most valued item was the attention of the nursing professionals. The least valued items were the waiting time for the appointment and the time spent in the waiting room on the same day. The incidence of complications recorded during the test were minimal (2% of all patients).

**Conclusions:**

Most patients are satisfied with the sedation administered by nurses via digestive endoscopies, and complications were rare, supporting the efficacy and acceptance of this practice. Clinical practice guidelines and consensus documents in Spain guarantee that nurses have autonomy to administer sedation in digestive endoscopy services, but there is a lack of national regulations to support this technique. The current consensus in Spain is that specific training is necessary for such nurses.

## Introduction

Gastrointestinal (GI) endoscopy is an essential technique for the diagnosis and treatment of diseases of the digestive system. This method significantly reduces the incidence and mortality of cancers such as colorectal and gastric cancer [1, 2]. As an invasive procedure, however, it can cause significant discomfort. While the use of sedatives such as propofol has improved patient comfort, certain barriers still hinder its widespread adoption. These include concerns about sedation and its side effects, as well as anxiety about the diagnosis and the procedure itself [3]. These concerns, which can increase the use of sedatives and complicate the procedure, underscore the importance of proper evaluation and management of patient anxiety before gastrointestinal endoscopy [4].

Sedation has become a standard practice in digestive endoscopy, ensuring both patient comfort and procedural effectiveness. In this context, the role of the nurse in the administration of sedation is crucial, but it also has specific challenges and responsibilities [5]. Traditionally, sedation was administered by anesthesiologists, but the increasing demand for endoscopic procedures has led to a shift, with nurses increasingly assuming this role [6]. This shift underscores the growing need for specialized training to ensure nurses administering sedation in digestive endoscopy acquire the necessary skills, especially in the environment of digestive endoscopy [7]. According to Sneyd [8], another important aspect is the legal framework that constrains the practice of sedation by nurses.

The role of the nurse anesthetist has evolved significantly, playing a crucial role in the administration of anesthesia and pain management [9]. The nurse anesthetist is an advanced practice nurse who, after specific training and certification, is trained to provide anesthesia in a variety of clinical settings [10]. Their responsibilities encompass pre-procedural assessments, intra-procedural care, pain management, and post-anesthesia recovery, ensuring patient safety throughout the process [10]. Nurse anesthetists work in diverse settings, including hospitals, ambulatory surgical centers, and clinics, providing care for a wide range of procedures across specialties such as gastrointestinal, cardiac, and trauma surgeries [9, 10].

In the case of Spain, the competences and functions of anesthesia nurses were defined by the Spanish Association of Anesthesia, Resuscitation and Pain Therapy Nurses (ASEEDAR-TD) as those of a specialist nurse [11]. These competencies of nurse anesthetists in Spain coincide to a large extent with those defined by the International Federation of Nurse Anesthetists [12]. Currently, different universities in Spain offer postgraduate training with a Master’s degree in the field of anesthesia, resuscitation and pain therapy. However, although the speciality is not recognised, there is a large number of nurses working in the field of anesthesia and their functions and competences include pre-anesthesia assessments, perioperative nursing, sedation outside the surgical area, resuscitation units, acute pain units and chronic pain units, coinciding with the role of a nurse specialist [12, 13].

Nurse prescribing in Spain is regulated and does not include the drugs needed for sedation. Some scientific societies (especially those in Gastroenterology) have developed their guidelines/protocols, unlike those referring to anesthesiology. Training is also not regulated in Spain, which is a pending issue, as there are established criteria by the International Council of Nurses and the International Federation of Nurse Anesthetists [11–13].

Nurses’ interventions in this context go beyond the simple administration of drugs, encompassing preprocedure evaluation, continuous monitoring and management of possible complications, and control of pain and sedation during the procedure [14, 15]. This is because acute pain can trigger an inflammatory response to stress, negatively affecting the patient’s progression [16]. In fact, current clinical practice guidelines recommend prioritizing sedation strategies based on analgesia and the administration of minimal doses of sedatives, with the aim of maintaining superficial or conscious sedation [17].

Safety and patient satisfaction are key metrics for assessing the quality of digestive endoscopy services. Regarding safety, ensuring minimal complications associated with nurse-administered sedation. Serious complications are relatively rare but require a high level of preparation and rapid response by the nurses involved [18]. The complications include risks of cardiopulmonary events such as hypoventilation, respiratory depression, apnea, hypotension, and bradycardia [19]. In this sense, sedative medications can cause muscle relaxation, compromising the ability to keep the airways open, while painkillers, especially opioids, can slow breathing. These complications can lead to hypoxemia, which if not detected and corrected in time can result in permanent damage to vital organs or even death [20].

Patient satisfaction is another crucial metric of the quality and successful nurse-administered sedation. Patient satisfaction is measured through post-procedure surveys, assessing aspects such as comfort during sedation and the quality of information provided before and after the procedure. Patients receiving sedation by nurses span a broad spectrum, from individuals with chronic conditions, including chronic diseases such as diabetes or heart disease, to acute cases. These patients may require sedation for a variety of procedures [21, 22]. Patients sedated by competent nurses report high levels of satisfaction and low perceived pain during the procedure [23]. Hoshijima et al. [24] reported that sedation-induced amnesia may be a contributing factor to greater patient satisfaction. In fact, it is recommended that nurses invest adequate time in offering personalized verbal advice, adapted to the educational level of the patient, to reinforce their understanding before undergoing an endoscopic procedure [25].

As we can see, sedation by the nursing staff play a central role in both maintaining safety and achieving high satisfaction levels. One of the most frequently performed diagnostic and therapeutic tests requiring sedation are digestive endoscopies. In Spain, although the nurse usually performs sedation in endoscopy services, few studies have been carried out in a representative sample to analyze the satisfaction of these patients with the care received and safety in the administration of sedation by nursing staff [26, 27]. Therefore, it is necessary to know the satisfaction of patients and safety with the administration of sedation in digestive endoscopies in a representative sample.

The main objective of this study was to determine the levels of patient satisfaction with the practice of sedation by the nurses in digestive endoscopies. The secondary objectives were to determine the safety of sedation practice by analyzing complication rates and doses used for sedation in endoscopic procedures.

## Materials and methods

### Design

A cross-sectional study was carried out at the Digestive Endoscopy Service at the University Hospital of Toledo from June to September 2023. A cross-sectional study is the optimal design for measuring patient satisfaction and safety with sedation in digestive endoscopy because it captures an accurate snapshot of current opinions, ensuring clinical relevance and direct applicability. Because it is cross-sectional, it collects data from a representative sample of patients over a short period of time, providing generalizable insight into nurses’ perceptions of sedation quality.

The study was conducted following the Strengthening the Reporting of Observation Studies in Epidemiology (STROBE) checklist [28]. The data collection period was June–September 2023.

## Participants/sample

The reference population was adult users over 18 years of age who attended the digestive endoscopy service of Toledo University Hospital to perform endoscopies with sedation.

The inclusion criteria were as follows:

- User of the digestive endoscopy service of the University Hospital of Toledo during the period of data collection.
- Patient undergoing digestive endoscopy with sedation.
- Patient with status I-II according to the ASA (American Society of Anesthesiologisth Physical) classification [29].
- Patient with status I-II according to the Mallampati classification [30].
- Patient undergoing any of the following endoscopic techniques: gastroscopy, colonoscopy, echoendoscopy or gastrocolonoscopy.

The exclusion criteria were:

- Pediatric patient or dependent on the pediatric service, under 18 years of age.
- Patient undergoing endoscopic technique: endoscopic retrograde cholangiopancreatography.
- Patients with problems reading the information sheet and signing the informed consent to participate in the study, such as mental impairment, illiteracy or incapability to read and understand the language.

The study was carried out at the University Hospital of Toledo, located southeast of the city of Toledo (Spain). It is a third level hospital inaugurated in 2020, designed to offer advanced and accessible healthcare services, with technologically equipped facilities and ample space for medical care.

The study area was the endoscopy service of Toledo University Hospital. In this service, endoscopies are performed (gastroscopies, colonoscopies, and digestive echo endoscopies) with sedation by the endoscopy staff only on those over 18 years of age. Therefore, the study population was all legal adults.

The sampling was carried out with the GRANMO program for population estimation, taking as a reference the previous study by Veldhuijzen et al. [31], in which users reported a total satisfaction score of 79.6 out of 100 points. Therefore, a random sample of 278 individuals would be enough to estimate, with a confidence of 95% and a precision of ± 5% points, a population percentage that will foreseeably be around 79.6%. A replacement rate of 10% was established. However, to ensure the representativeness of the sample, 660 people finally participated. Consecutive sampling was carried out until the desired sample was reached.

### Variables

The following independent variables were collected: age, sex, weight, height, allergies, abdominal surgeries, important diseases and/or diseases of interest, habitual medication, consent, pre and post vital sign monitoring, ASA classification, and Mallampati classification.

In turn, the following variables related to patient satisfaction with the service were analyzed (collected through the GHAA-9 m questionnaire): waiting time for the test, care provided by the medical staff, care provided by the nursing staff, quality of the information received, and discomfort during the test.

### Instruments

The data collection instruments used were the medical records of the patients and a satisfaction questionnaire that the users completed. The specific instruments were the following:

- *Digestive endoscopy service satisfaction survey.* We used the validated Spanish version [32] of the questionnaire on patient satisfaction with gastrointestinal endoscopy put forth by the American Society of Gastrointestinal Endoscopy (GHAA-9 m) [33]. This survey evaluated the quality and results of gastrointestinal endoscopy. This survey evaluated the quality and results of gastrointestinal endoscopy. The scale consists of nine items, seven of which are measured on a Likert scale of 1 to 5 where 1 is bad and 5 is excellent, and two others are measured on a yes or no scale.
- *Endoscopy control and treatment sheet.* This tool is commonly used in the endoscopy service of the University Hospital of Toledo. This document collects sociodemographic data of the patient, such as sex and age, as well as information about pathologies and diseases that must be taken into account to carry out the technique safely and effectively, such as lung, heart, kidney disease, intervention procedures, allergies and pharmacological treatment. It also collects the medication and dose administered for sedation. This sheet also contains a section where some constants, such as oxygen saturation (SatO_2_), blood pressure (BP), and heart rate, are collected (HR) before sedation, during sedation, and after sedation, as well as levels of consciousness, recovery after sedation, and incidents. Finally, this sheet also contains the assessment of the following scales:
  - *Classification of the ASA physical status.* This estimates the risk posed by anesthesia for the different states of the patient. It was created by a committee consisting of Meyer Saklad, Ivan Taylor, and Emery Rovenstine [34]. This scale is scored from 1 to 6; patients scoring 3 or more are those who present a certain risk: these are people who have a serious illness. Patients classified as 4 or 5 had severe disease and might not survive more than 24 h without surgical intervention. Patients classified as 6 had declared brain death, and their organs were removed for donation [29, 35].
  - *Aldrete scale.* It was created by Dr. Aldrete [36]. It is an evaluation scale used to assess patients undergoing general anesthesia and determine their transfer after the immediate recovery of the patient. This scale consists of 5 items. Each item is rated on a Likert-type scale from 0 to 2, with a total range that ranges from 0 to 10. The cutoff point is 9, where a score equal to or greater than this value suggests adequate recovery after anesthesia.
  - *Mallampati classification.* Designed by Mallampati [30], this scale or classification is used to predict the ease of intubation. It is assessed by analyzing the anatomy of the oral cavity. It is based on the visibility of the base of the uvula, the isthmus of the jaws, and the soft palate. A score is determined by the manifesting or not of phonation. A high score (class 4) is associated with difficult intubation, as well as a high incidence of sleep apnea.

These scales estimate the risk of anaesthesia for patients and help to assess whether sedation can be performed by the endoscopy nurse. The nurse anaesthetist can only administer sedation in ASA I and II and Mallampati I and II patients. In case of higher assessments, sedation should be administered by an anaesthetist.

The data on this sheet do not include patient identification; only anonymized clinical data were used.

### Procedure

Patients were recruited by the nurses of the endoscopy service. The patients who met the inclusion criteria were provided with the information sheet by the nurse and signed the informed consent form if they wished to participate in the study. Participants were added to the study consecutively until the sample was complete. After the consent was signed and the examination was completed and before discharge, a questionnaire was provided for completion. This questionnaire included a survey of patient satisfaction with the digestive endoscopy service.

The remaining digital clinical history of the patient corresponding to the endoscopy control and treatment sheet was obtained, maintaining the anonymity of these patients and using only clinical data corresponding to the performance of the endoscopic technique and clinical history of interest.

### Statistical analysis

For the statistical analysis, SPSS version 28 program, which is licensed by the University of Castilla-La Mancha, was used. The qualitative variables are expressed as counts (n) and percentages (%). The quantitative variables are expressed as the arithmetic mean (m) and standard deviation (SD). An inferential analysis was performed to determine the relationships of the independent variables with the dependent variables:

For qualitative variables, proportions were compared between groups using the chi-squared test for contingency tables. For 2 × 2 tables, the chi-squared test with Yates’s correction was used, and when any expected frequency was ≤ 5, Fisher’s exact test was applied.

For the quantitative variables, first the goodness of fit to a normal distribution was determined by the Shapiro‒Wilk test, as was the homogeneity of the variances by the Levene test. As the data fit a normal distribution, Student’s t test was used.

All hypothesis tests are bilateral. In all the statistical tests, statistically significant values were those whose confidence level was 95% (*p* < 0.05).

### Ethical considerations

This study was approved by the Ethics Committee for Clinical Research with Medicines of the “Complejo Hospitalario Universitario de Toledo” on 5/5/23 n° 1012. All participants read the information sheet and signed informed consent to participate in the study. The research respected the fundamental principles of the Helsinki Declaration, the Council of Europe Declaration on Human Rights and Biomedicine, the UNESCO Universal Declaration on the Genome and Human Rights and the Oviedo Council on Human Rights and Biomedicine. All participants’ data were treated confidentially in accordance with the Organic Law 3/2018, of 5 December, on Personal Data Protection and guarantee of digital rights.

## Results

### Description of sample and type of endoscopy

A total of 660 patients were included in the study, 347 women (52.6%) and 313 men (47.4%). The average age of the participants was 58.24$\:\pm\:$ 13.4 years (range 18–89 years). Different endoscopic examinations were performed—gastroscopy (31.4%), colonoscopy (61.5%), echo endoscopy (3%) and gastro-colonoscopy (4.1%)—with significant relationships between the type of examination and sex (Table 1).

**Table 1:** Distribution of patients by type of endoscopic examination and sex

Endoscopic examination type	Women *n* (%)	Men *n* (%)	Total *n* (%)	*p*-value χ2
Gastroscopy	131 (63.3%)	76 (36.7%)	207 (31.4%)	< 0.001
Colonoscopy	186 (45.8%)	220 (54.2%)	406 (61.5%)	
Echoendoscopy	12 (60%)	8 (40%)	20 (3%)	
Gastrocolonoscopy	18 (66.7%)	9 (33.3%)	27 (4.1%)	

### Previous pathologies of the patients and complications

Regarding the pathologies and comorbidities of the patients treated, the following stood out: previous intervention involving abdominal surgery (32%), diabetes (16.2%), heart disease (12.9%), lung problems [such as chronic obstructive pulmonary disease (COPD) or obstructive sleep apnea syndrome] (5.6%), and kidney problems (2.4%). In fact, 65.3% of the participants took medication daily.

The incidence of complications recorded during sedation was 13 (2% of all patients). Among them were desaturation, bradycardia, hypotension, and vomiting, which were easily resolved by the nurses by opening the airway, placing the patient in a lateral safe position, and administering atropine or ephedrine.

There were no significant differences between patient satisfaction and previous pathologies (0.318) or the incidence of complications (0.322).

### Doses and drugs most commonly used

Regarding the doses and drugs used, 100% were administered as a single drug, 1% propofol, with a total average dose of 199.51 mg (SD ± 83.4) administered by a continuous infusion pump through a peripheral venous catheter. The guidelines for the induction, maintenance, and total dose of propofol in each of the different procedures are described in Table 2. The total dose administered differed by endoscopic procedure: being gastrocolonoscopy the endoscopic technique that required the highest total dose of 1% propofol (272.96 ± 90.588) and gastroscopy the one that required the lowest dose (154.88 ± 56.613) (*p* < 0.001).

**Table 2:** The dose of propofol was 1% for the induction and maintenance of sedation used for each of the different endoscopies

		*n*	Medium	Standard deviation	Min	Max	*P*
Propofol induction regimen	Gastroscopy	207	197.10	16.079	100	200	0.750
	Colonoscopy	406	196.06	19.481	100	200	
	Echoendoscopy	20	200.00	0.000	200	200	
	Gastrocolonoscopy	27	196.30	19.245	100	200	
Propofol maintenance guideline	Gastroscopy	207	36.49	7.735	16	60	0.257
	Colonoscopy	406	35.78	6.976	10	82	
	Echoendoscopy	20	38.70	8.498	20	60	
	Gastrocolonoscopy	27	35.70	6.082	25	50	
Total Propofol dose	Gastroscopy	207	154.88	57.613	60	460	< 0.001
	Colonoscopy	406	215.67	83.284	40	600	
	Echoendoscopy	20	234.30	99.701	120	510	
	Gastrocolonoscopy	27	272.96	90.588	110	460	

Student’s t test was used

We monitored the patients’ vital signs (BP, HR, and SatO_2_). Supplemental O_2_ (4–6 L/min) was administered through nasal glasses. All the patients had a complete recovery, with a score of 10 on the Aldrete scale, and there was no adverse event. Table 3 shows the monitoring of the constants, with significant differences (*p* < 0.001) pre- and postsurgery.

**Table 3:** Vital sign monitoring

	Minimum	Maximum	Mean ± SD	*p*
Pre systolic BP	88	214	136.59±20.924	< 0.001
Post systolic BP	56	763	124.13±31.711	
Pre diastolic BP	41	157	82.60±13.856	< 0.001
Post diastolic BP	42	145	76.40±12.643	
Systolic BP D1	71	745	128.88±31.790	
Systolic BP D2	50	204	124.58±20.527	<0.001
Systolic BP D3	75	210	124.35±19.440	0.361
Diastolic AT D1	40	138	78.94±13.803	
Diastolic AT D2	36	136	77.98±12.926	0.045
Diastolic AT D3	36	170	77.33±12.706	0.093
Pre heart rate	40	152	77.87±14.502	< 0.001
Post heart rate	38	125	70.82±11.578	
Heart rate D1	33	120	74.15±12.807	
D2 heart rate	40	129	72.38±12.347	0.444
D3 heart rate	40	120	71.48±11.882	0.023
Presedation saturation	90	100	99.31±1.433	< 0.001
Postsaturation duration	93	100	99.75±0.790	
Saturation D1	90	100	99.60±1.112	
Saturation D2	93	100	99.68±0.945	0.009
Saturation D3	92	100	99.68±0.920	0.438
Total score Aldrete Scale	10	10	10.00±0.000	-

Student’s t test was used

### Patients’ satisfaction

Regarding the satisfaction of the patients, this is shown in Tables 4 and 5, if we look at each of the questions contained in the survey, we can see that the worst-valued item was the waiting time for the appointment and the time waiting in the exam room the day of the appointment. The most highly rated item was the care provided by nursing professionals, followed by the care provided by medical professionals (Table 5).

**Table 4:** Responses of the participants to the different items of the digestive endoscopy service satisfaction scale

Item	Bad *n* (%)	Normal *n* (%)	Good *n* (%)	Very good *n* (%)	Excellent *n* (%)
Appointment waiting time	33 (5%)	78 (11.8%)	180 (27.3%)	175 (26.5%)	194 (29.4%)
Time to wait on the day of the test	15 (2.3%)	60 (9.1%)	122 (18.5%)	217 (32.9%)	245 (37.1%)
Medical personal care	0	0	38 (5.8%)	177 (26.8%)	445 (67.4%)
Nursing personal care	0	0	25 (3.8%)	145 (22%)	490 (74.2%)
Explanations about the test	0	1 (0.2%)	63 (9.5%)	218 (33%)	378 (57.3%)
Discomfort during the test	0	14 (2.1%)	52 (7.9%)	182 (27.6%)	412 (62.4%)
Overall score	1 (0.2%)	9 (1.4%)	70 (10.6%)	233 (35.3%)	347 (52.6%)
	Yes			No	
I would come back to the same hospital	658 (99.7%)			2 (0.3%)	
I would come back to the same professionals	660 (100%)			0	

**Table 5:** Responses of the participants to the different items of the digestive endoscopy service satisfaction scale for each type of test

Appointment waiting time						Waiting time on the day of the test					Medical personal care					Nursing personal care				
Scan type	Bad	Normal	Good	Very good	Excellent	Bad	Normal	Good	Very good	Excellent	Bad	Normal	Good	Very good	Excellent	Bad	Normal	Good	Very good	Excellent
	%	%	%	%	%	%	%	%	%	%	%	%	%	%	%	%	%	%	%	%
Gastroscopy	6.30%	12.10%	25.10%	28%	28.50%	4.30%	7.20%	19.80%	30.90%	37.70%	0	0	6.30%	26.10%	67.60%	0	0	3.90%	21.30%	74.90%
Colonoscopy	4.90%	11.60%	29.60%	24.40%	29.60%	1.50%	9.40%	18.50%	34.10%	36.50%	0	0	5.20%	27.80%	67%	0	0	3.40%	23.60%	72.90%
Echoendoscopy	0	15%	25%	40%	20%	0	20%	15%	40%	25%	0	0	15%	25%	60%	0	0	10%	20%	70%
Gastrocolonoscopy	0	11.80%	11.10%	37%	40.70%	0	11.10%	11.10%	25.90%	51.90%	0	0	3.70%	18.50%	77.80%	0	0	3.70%	3.70%	92.60%

	Explanations about the test					Discomfort during the test					Overall score					I would repeat in the same hospital		I would repeat with the same professionals	
Scan type	Bad	Normal	Good	Very good	Excellent	Bad	Normal	Good	Very good	Excellent	Bad	Normal	Good	Very good	Excellent	Si	No	Si	No
	%	%	%	%	%	%	%	%	%	%	%	%	%	%	%	%	%	%	%
Gastroscopy	0	0	8.70%	35.70%	55.60%	0	1%	5.80%	27.50%	65.70%	0	1%	9.20%	35.70%	52.20%	100%	0	100%	0
Colonoscopy	0	0.20%	9.60%	32.30%	57.90%	0	2.70%	8.90%	28.80%	59.60%	0	1.70%	11.60%	34.20%	52.20%	99.5	0.50%	100%	0
Echoendoscopy	0	0	20%	40%	40%	0	0	10%	25%	65%	0	0	15%	35%	50%	100%	0	100%	0
Gastrocolonoscopy	0	0	7.40%	18.50%	74.10%	0	3.70%	7.40%	11.10%	77.80%	0	0	3.70%	33.30%	63%	100%	0	100%	0

Regarding the waiting time for the different endoscopic examinations, the worst-valued test was gastroscopy, represented by 6.3% as “bad” and 12.1% as “normal”. Endoscopy was the best, with 40% “excellent”.

Regarding the discomfort perceived during the test, colonoscopy and gastrocolonoscopy were the worst, with 2.7% and 8.9% of patients experiencing discomfort perceived as “normal”, respectively, and the best value was for echoendoscopy, with 100% of patients experiencing positive responses.

## Discussion

In our study, the vast majority of patients were satisfied with the sedation administered by nurses in digestive endoscopy. Patient satisfaction with digestive endoscopy services with sedation administered by nurses is positive, as has also been shown by other previous studies carried out by has been found to be positive, as in previous studies [24, 25, 27, 37, 38]. Although these studies were carried out in other countries and had different geographical distributions and used different tools, the results were similar, so it would be important to implement universal tools to make comparisons. This high patient satisfaction with the technique applied by nurses may reflect the efficacy and acceptance of the practice of sedation administered by nurses. These results align with those of Minciullo and Filomeno [39], who reviewed patient satisfaction in digestive endoscopy and obtained the same results: patient satisfaction and a low number of acceptable complications.

However, there are studies that have applied new technologies, such as the use of educational videos, to improve patient satisfaction in digestive endoscopies. In fact, recent studies have shown that patients who viewed videos were more satisfied than patients who only received standard information. These results suggest that educational videos can be an effective tool for improving patient satisfaction with endoscopy [40]. Another study implemented virtual reality in children between 5 and 16 years old who underwent upper-digestive endoscopies, reducing pain by the use of virtual reality and registering a high degree of satisfaction [41].

Though patient satisfaction is a crucial indicator of quality of care, it is influenced by numerous factors, including personal and contextual factors such as bowel preparation for colonoscopy, hours of fasting, therapeutic procedures, the performance of the techniques by residents and/or students, the degree of baseline anxiety, the lack of knowledge, and the unsafe environment, among others [42]. Patient satisfaction is also closely related to waiting times, both waiting for the day of the appointment and waiting on the day of the procedure itself, as was the case in similar studies [26, 31, 42, 43]. Long times lead to a lower satisfaction that can affect the overall assessment. In this sense, the adoption of measures to solve these problems should be valued, such as working on the management of agendas.

Regarding safety, the anesthetic of choice was 1% propofol, with minimal complications (2%). The most common complication being transient desaturation, similar to others [37], where the percentage of complications was slightly higher at 3.05% and the most common complication was also transient desaturation and lower than others [44, 45], where the percentage of complications was 16%. With these data we can indicate that the practice of nurse sedation is a safe practice, as also indicated by other studies [46].

Regarding the use and doses of propofol, there are various methods for administering propofol for sedation by nurses in endoscopic procedures that depend on the duration of the examination, the complexity of the examination, and the personnel available in the unit [44]. In our study, the only anesthetic used was 1% propofol, with differences between the doses administered according to the procedure. These dose variations are influenced by multiple factors, including patient characteristics (age, weight, comorbidities due to associated diseases), the complexity of the procedure, and institutional policies. This percentage is above that of some studies and below that of others [37]. The use of other types of drugs in combination with propofol, such as midazolam and lidocaine, does not indicate the type or complexity of the test performed or the characteristics of the patient, so it is difficult to compare. Some studies have used lower doses of propofol in combination with other sedatives or analgesics to achieve a synergistic effect [5]. This practice seeks to reduce the side effects associated with higher doses of propofol, such as respiratory depression. The choice of sedative and the depth of sedation must be carefully considered to maximize patient satisfaction. Other studies indicate that the use of alternative drugs to propofol, such as fospropofol and ciprofol, show promise [2]. Fospropofol offers a slower onset of action and less pain during application, while ciprofol is characterized by a rapid onset of action and a lower incidence of pain at the injection site. Additional studies are needed to determine the most recommended doses and anesthetics needed to improve patient safety and comfort.

Continuous monitoring of the patient during sedation is essential to adjust the dosage of propofol and to intervene quickly in cases of complications, such as respiratory or hemodynamic depression. The experience and training of the nursing staff who administer propofol are crucial to ensure safe and effective sedation. Nurses should be trained in the management of respiratory and cardiovascular emergencies associated with sedation [2]. However, the role of nursing in endoscopy sedation has been widely studied and documented in the literature. Nurses play a crucial role in the administration and monitoring of sedation, ensuring the safety and comfort of patients during the procedure. In clinical practice, nurses are responsible for assessing the patient’s condition before the procedure, preparing and administering sedative medications, and constantly monitoring vital signs and the patient’s response to sedation [46, 47]. The training and experience of nurses in the administration of sedatives and in the management of possible complications are vital for the success of endoscopic procedures. In fact, the Spanish Association of Digestive Endoscopy provides doctors and nurses of digestive endoscopies with the necessary training through courses that it organizes in different Spanish hospitals throughout the year [44]. The participation of nurses in the recovery phase of patients is also a fundamental aspect of their role. They monitor the patient’s recovery from sedation by evaluating their ability to return to normal activities [39, 48–51].

As implications for clinical practice, this study has shown that patients are satisfied with the sedation offered by the nurses of the digestive endoscopy service. This can help to improve the quality of care and patient safety, since, as we have seen, the complications of the procedure were minimal. Likewise, this satisfaction also helps nurse-patient communication, which can lead to greater trust and cooperation. Likewise, knowledge of sedation techniques can help in designing interventions to reduce anxiety. Likewise, efficient sedation administered by nursing staff can reduce the need for more specialized staff and free up resources. Finally, further progress is needed to create regulations and policies to regulate the practice of sedation by nurses and to advance the specialty of nurse anesthetists.

### Limitations

This study has some limitations. First, it had a cross-sectional design, so it cannot establish causal relationships. Second, regarding the age of the participants, older people sometimes found it difficult to fill out the questionnaire. In those cases, the nurse helped them understand all the items. Also external conditions such as physical environment, interaction with other members of the healthcare team or waiting time may influence satisfaction without being directly related to service quality. Finally, one possible limitation was the self-reported information.

As the main strength of our study, we can highlight the large and representative sample (more than 650 patients).

## Conclusions

User satisfaction with the sedation done by nurses for endoscopy was high. This indicates that patients are satisfied with the service received. The drug used for sedation was 1% propofol in all the examinations and there were minimal complications (2%). Regarding the diversity of patients treated, they had different comorbidities, and a high percentage took medication daily. Based on the results, sedation administered by nurses in the endoscopy service is a safe and effective technique since there are few complications.

Future studies should be done in different geographical areas with the same tools to globally assess patient satisfaction. Such studies would allow us to detect opportunities for improvement, seeking solutions to the problems encountered and therefore improving the quality of services.

Patient satisfaction is one of the key pillars of quality care management programs. It should be taken into account that satisfied patients better comply with the indications and follow-up and better tolerate the different treatments, while patients dissatisfied with the care received may suffer anxiety and stress, making them not respond fully or correctly to the proposed treatments.

This study highlights the satisfaction of patients with sedation by nursing staff. Although no European or international regulations regulate the competences of the administration of sedation by nurses in digestive endoscopy services, some recommendations and quality standards have been developed by different European scientific organizations and societies, such as the European Society of Gastrointestinal Endoscopy and the European Society of Gastroenterology and Endoscopy Nurses and Associates. These recommendations and standards address aspects such as the training and competence of personnel, the criteria and procedures for sedation, the monitoring and safety of patients, and the documentation and recording of data. The application of such recommendations depends on the legislation and practice of each member country of the European Union. Therefore, formulators of health policies must write specific legislation so that nurses can administer sedation in this service legally internationally.

## Data Availability

The data used to support the findings of this study are available from the corresponding author upon request.
